# The Impact of Popular Science Articles by Physicians on Their Performance on Online Medical Platforms

**DOI:** 10.3390/healthcare10122432

**Published:** 2022-12-02

**Authors:** Jingfang Liu, Shiqi Wang, Huihong Jiang

**Affiliations:** School of Management, Shanghai University, Shanghai 201800, China

**Keywords:** online healthcare, popular science articles, physician performance, elaboration likelihood model

## Abstract

The public demand for popular science knowledge regarding health is increasing, and physicians’ popular science practices on online medical platforms are becoming frequent. Few studies have been conducted to address the relationship between specific characteristics of popular science articles by physicians and their performance. This study explored the impact of the characteristics of popular science articles on physicians’ performance based on the elaboration likelihood model (ELM) from the central path (topic focus and readability) and the peripheral path (form diversity). Data on four diseases, namely, lung cancer, brain hemorrhage, hypertension, and depression, were collected from an online medical platform, resulting in relevant personal data from 1295 doctors and their published popular science articles. Subsequently, the independent variables were quantified using thematic analysis and formula calculation, and the research model and hypotheses proposed in this paper were verified through empirical analysis. The results revealed that the topic focus, readability, and form diversity of popular science articles by physicians had a significant positive effect on physicians’ performance. This study enriches the research perspective on the factors influencing physicians’ performance, which has guiding implications for both physicians and platforms, thereby providing a basis for patients to choose physicians and enabling patients to receive popular science knowledge regarding health in an effective manner.

## 1. Introduction

In recent years, with the progress of internet technology, the strong support of relevant national policies, and the injection of social capital, internet healthcare has developed rapidly and online medical platforms have gradually been recognized by the public [1]. The online medical platform is a new application of the internet in the medical industry that can provide users with online medical treatment, health information, appointment registration, and other services and can effectively alleviate the unequal distribution of medical resources and urban–rural health disparities [2,3]. With the increasing numbers of doctors on platforms, patients are able to consider factors such as a doctor’s title and hospital grade. In addition to the characteristics based on individual physician attributes, patients may be more inclined to gain additional information about the doctor through other means to increase trust in them so that they can find a doctor who may be suitable for them in a more targeted manner.

Online healthcare platforms facilitate doctor–patient interaction, and physicians play a significant role in the success of online health communities (OHCs) [4]. Through online consultations, doctors can generate actual income and improve their performance. If doctors can achieve higher performance on online platforms, they will be motivated and more serious about continuing to provide online medical services, thus, leading to a boom in such platforms. Online medical platforms allow patients to communicate with doctors online in the form of graphics, phone calls, or videos by paying for consultations online. The doctor’s professional competence promotes the patient’s perceived trust in the doctor, and the doctor’s integrity and kindness enable the patient to perceive emotional trust [5]. Information and emotional support provided by physicians during doctor–patient interactions can enhance patient satisfaction [6]. The previous research was primarily based on the patients’ perspective, exploring factors influencing patients’ choice of physician and their satisfaction [7,8,9]. The sustainability of online healthcare platforms is also dependent on the contributions of physicians. However, physicians remain unsure on how active participation can best improve their online performance. The current research on physicians’ performance focuses on objective data obtained from physicians [10]. For example, Liu, Guo, Wu, and Wu verified that physicians’ offline and online reputations and the online and offline ratings of hospitals where physicians are employed were positively associated with their performance [11]. The factors influencing physician team performance from a team perspective were studied [12,13]. Wang, Liu, and Ye explained the effect of individual and team specialized capital on physicians’ performance based on a cross-level model and revealed that physicians’ status capital and decision capital were positively related to their performance [14].

The above studies reveal that the findings of previous studies have mostly involved the effect of physicians’ personal characteristics on their performance, while ignoring other ways in the platform that can improve performance. This study focuses on physician-posted popular science articles as the subject of the study to investigate the role of physician-posted popular science articles on physicians’ performance.

Popular science articles are a type of article that disseminate popular science knowledge from the perspective of ordinary people. Parkinson and Adendorffn found that popular science articles can make science more accessible to students during science instruction [15]. Furthermore, in recent years, the term “science popularization” is also widely used in the online medical field. There is a serious information asymmetry between doctors and patients in the medical field; as patients have limited medical expertise, doctors often dominate the doctor–patient interaction. With the development of internet healthcare and the increase in health awareness among the public, more and more doctors will disseminate popular science knowledge to everyone online, thus, realizing health knowledge sharing between doctors and patients. Physician quality and the quality of Internet health information have a significant impact on physician–patient alignment, so physicians should make their patients aware of their professional capabilities as much as possible [16]. The study found that the act of health literacy by physicians not only helped patients to learn more about the subject but also improved their own professional competence [17]. In addition to providing online medical services, online medical platforms also serve an important function in health knowledge popularization. As online health information becomes more and more popular among patients, patients will browse more frequently on online medical platforms, and consequently, doctors will publish more and more popular science articles on such platforms [18]. Research related to popular science articles is mainly divided into the antecedents and consequences of publishing popular science articles, i.e., the motivation of sharing science articles and utility of science articles. Studies have found that online popular health science knowledge enables patients and their relatives to receive information support [19,20]. Zhang, Liu, Deng, and Chen distinguished between general users and medical professionals to explore their motivations for sharing online health knowledge [21]. Research on the utility of health articles shows that doctors can benefit from sharing their health knowledge with patients through online medical platforms. Patients who gain health knowledge from a doctor’s popular science articles will have trust in the doctor and will be more likely to choose the doctor for future consultation services, increasing the doctor’s financial return [22]. Moreover, studies on the characteristics of popular science articles have revealed that the completeness and readability of the content of popular science articles can positively influence the acquisition of health knowledge by patients and that the acquisition of health knowledge contributes to patients’ self-efficacy [23].

Based on the aforementioned literature review, we found that most of the current studies on popular science articles have focused on the motivation of physicians to practice popular science and the factors influencing patients’ willingness to adopt health information. There are a few studies on the characteristics of popular science articles; the relationship between popular science articles and physicians’ performance has focused solely on the characteristics of quantity and reading volume. This study investigates the impact of specific characteristics of popular science articles on physicians’ performance in relation to physicians’ published articles.

The features of popular science articles can be divided into surface and content features. The format of the article (text, voice, and video) is a superficial feature. By observing the variety of formats presented by doctors’ popular science articles, patients can judge how active and attentive doctors are on online platforms and may be more inclined to trust such doctors. Content features refer to the features that patients can judge by reading the text of popular science articles. The topics covered in articles are used to study whether patients prefer doctors who publish articles on a variety of topics or doctors who write targeted articles. The more readable the content of a popular article, the more likely it is to be accepted by patients owing to their lack of medical knowledge. Therefore, the goal of this study is to investigate which features of science articles promote physicians’ performance from the physicians’ perspective in terms of surface and content features.

## 2. Research Hypothesis and Theoretical Basis

### 2.1. Research Hypothesis

#### 2.1.1. Topic Focus

The topic is a key factor in understanding an article’s content, and the probability of distribution of topics can be identified by machine learning techniques [24]. In the e-commerce field, by analyzing the thematic characteristics of the review content, we can summarize the importance that consumers attach to different features of the product, thereby helping merchants attract more potential consumers [25]. In OHCs, analyzing the content topics posted by users in the community and mastering the topics that users are interested in can be more conducive to the dissemination and popularization of online health knowledge [26]. In this study, we assume that the more targeted the topic of the popular science article published by the physicians, the better is their performance. The reasons are as follows: in the medical field, each doctor has their own area of expertise, and the social and economic rewards for doctors are largely related to their professional competence [27]. The topic of popular science articles written by doctors can also be a factor for patients to judge doctors’ professional competence. If most of the popular science articles published by the doctor introduce health knowledge related to a specific disease or a specific field, it implies that the doctor has a strong grasp of knowledge in their related field, is more experienced, and is able to highlight their area of medical expertise; thus, patients are more convinced of the doctor’s professional competence. Therefore, based on the above points, we propose the first hypothesis:

**Hypothesis 1 (H1):**  *The topic focus of popular science articles has a positive impact on physicians’ performance.*

#### 2.1.2. Readability

Readability refers to the degree or nature of how simple a text is to read and is often used to measure the ease with which instructional materials for students can be understood [28]. Reading material is valuable when it has the right level of readability and conveys the intended information to the reader. The core criterion of readability is that the materials are adapted to the cognitive level of the students who use them, and when the two are matched, students’ learning will be better. Nowadays, the internet has become an important medium for disseminating information. The readability of internet information is an important criterion for measuring the accuracy of information conveyed [29]. As the requirements for accuracy and readability of health information are higher in the medical field than in other fields, if users cannot understand the content of health information, it can easily lead to misinterpretation by patients and other serious medical errors. Therefore, the readability of online health information is crucial. Related studies analyzed the readability of health information related to different diseases and found that the information exceeded the recommended reading level [30,31,32,33,34]. This can be difficult for the public to understand, and the science of health knowledge does not achieve the desired effect. If doctors on online medical platforms publish popular science articles that are difficult for patients to comprehend, patients will not obtain effective information to evaluate doctors’ professional competence, which, in turn, will greatly diminish doctors’ efforts. If doctors can consider patients’ ability to understand healthcare information, patients’ trust in doctors will increase, thereby helping to improve doctors’ performance. Based on the above, we propose a second hypothesis:

**Hypothesis 2 (H2):**  *The readability of popular science articles has a positive impact on physicians’ performance.*

#### 2.1.3. Form Diversity

With the booming development of new media, various forms of information are released on the internet. According to the richness of the media, three main media formats can be classified—text, voice, and video [35]. Users can relieve their emotional problems by conveying emotions via text interaction on the internet [36,37]. However, text communication cannot effectively meet the characteristics of timeliness, and when there is too much text, the reader may lose patience [38]. In information dissemination, expressions and gestures are also important factors for better understanding in readers, and videos further enhance the communication effect [39]. In summary, we determine that all three media forms—text, voice, and video—can play an important role in information dissemination. We found that media richness is positively correlated with user perception effect [40]. The greater the media richness of the platform or the individual providing the service, the greater will be the loyalty of the users [41]. The richer the variety of media used by physicians to publish popular science articles in the online medical field, the broader the range of different patient needs with increased ability to formally attract more patients and improve physicians’ performance. In summary, based on the above points, we propose a third hypothesis:

**Hypothesis 3 (H3):**  *The form diversity of popular science articles has a positive impact on physicians’ performance*.

**Figure 1:**
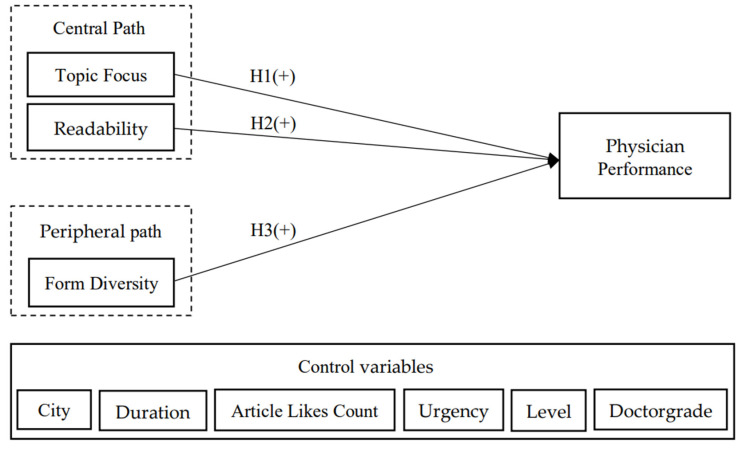
Research Model.

Based on these hypotheses, we propose the model for this study, as shown in Figure 1. The purpose of this paper is to investigate the influence of the content and form characteristics of physician popular science articles on physician performance. Based on the research concerning users’ perceptions of using the doctors’ science popularization area in online medical platforms and the summary of the previous literature on factors influencing information utility, combined with the special topic of health science knowledge, we finally extracted two content features: topic focus and readability, and one form feature: form diversity. Based on the ELM model, the characteristics of physician-published popular science articles were divided into a central path (topic focus and readability) and a marginal path (form diversity) according to the degree of fine processing to investigate whether topic focus, readability, and form diversity have a positive impact on physicians’ performance. Among them, based on the findings of existing studies on factors influencing physicians’ performance and the data we obtained on personal attribute characteristics related to physicians in online medical platforms, the control variables in the final model of this study include city level (the rank of the city where the physician is located), online service duration of the physician (the length of time the physician has been registered to the platform), number of likes on popular science articles (the average count of likes on physicians’ articles), degree of disease urgency (degree of need for timely treatment), hospital rank (whether the doctor’s hospital is a tertiary hospital), and doctor’s title (chief physician, deputy chief physician, and other physicians). We will also explore the impact of the above control variables on physician performance.

### 2.2. Elaboration Likelihood Model

The elaboration likelihood model (ELM), also known as the dual-path model, is a model that describes the process and pathways of consumer attitude change. The model suggests that the process of convincing individuals to change their attitudes has two paths of thinking: the central and peripheral paths [42]. Depending on the degree of in-depth information processing, an individual’s attitude may be persuaded by the central or peripheral path. The central path of persuasion will be more important when an individual has a higher degree of in-depth information processing. In this case, the change in an individual’s attitude depends mainly on the result of their perception after thinking deeply about the information. The peripheral path of persuasion is more important when an individual has a low level of in-depth information processing. In this case, the individual’s attitude is mainly determined by environmental factors and representational cues [43]. Additionally, previous research has shown that the ELM model for understanding information adoption can be extended to the knowledge management domain [44].

ELM is widely used in the field of information systems. Alpar, Engler, and Schulz used the ELM model to study users’ perceptions of the usefulness of reports generated by business intelligence software [45]. The authors determined that author rank and number of report subscriptions were peripheral cues to users’ concerns and that voting schemes were the central path to users’ perceptions of report usefulness. Wang and Yang examined the funding intention of backers in reward crowdfunding based on the ELM and deduced that the central (product innovativeness, perceived product quality, and creator competence) and peripheral paths (web visual design) had a positive impact on backers’ funding intention [46]. The ELM has been used to investigate the impact of information quality and source credibility on the willingness to adopt knowledge [47,48,49].

The ELM is also widely used in health scenarios. Cao and Liu et al. studied patient selection behavior using the ELM [50]. Service quality was considered the central pathway and online word-of-mouth was considered the peripheral pathway; the moderating impacts of disease risk and disease-related expertise on patients’ willingness to visit were examined. Disease risk increases the significance of service quality on patient choice, whereas disease risk and disease-related expertise decrease the importance of online word-of-mouth on patient choice. Zhang, Yan, and Cao et al. investigated how users’ e-health literacy influenced their willingness to continue using online health apps and found that the moderating effect of users’ e-health literacy was largely significant along the peripheral path [51]. Zhou verified that users’ willingness to adopt information would be affected by the central (quality of arguments) and peripheral paths (source credibility and emotional support) based on the ELM [52]. Moreover, self-efficacy can moderate the influence of the central and peripheral paths on the willingness to adopt information. Li, Zhang, and Han examined the factors influencing patients’ engagement in online follow-up services by considering the technical and interpersonal quality of physician care as the central pathway and online word-of-mouth as the peripheral pathway [53]. The authors demonstrated that all these factors positively influenced patients’ willingness to follow-up online.

In this paper, the fine processing likelihood model is used as the theoretical basis to explore factors influencing physicians’ performance in two ways. Physicians’ performance is used in the ELM as the result of choice after information processing. Patients can process information related to popular science articles in two ways and then consider whether to ask the physician for a consultation, i.e., whether it is beneficial to physicians’ performance. As the thematic relevance and readability of science articles require patients to develop their own conclusions after reading the articles, this study considers the thematic relevance and readability of science articles as the central path. Additionally, the diversity of science article formats is a representational cue, allowing patients to obtain the right answer without carefully reading the article contents; thus, this study considers the diversity of popular science article formats as the peripheral path. Both paths are used to study the impact of the characteristics of science articles written by physicians on their performance.

## 3. Method

### 3.1. Study Aim and Design

This study is based on data related to physicians’ published popular science articles and physicians’ personal tagging attributes from the scientific area of the online medical platform. We analyzed the impact on physicians’ performance in terms of the content and formal characteristics of popular science articles.

The study in this paper collected data from an online medical platform for empirical analysis to verify the research hypotheses. Based on the summary of existing literature, we selected LDA topic modeling and formula calculation methods to quantify the proposed variables and then analyzed the impact of content characteristics (topic relevance, readability) and form characteristics (form diversity) of science articles on physicians’ performance through mixed regression.

This study digs deeper into physician-published popular science articles to explore the role of their specific characteristics on physician performance.

### 3.2. Data Preparation

This study collected relevant research data from the online medical platform. This platform helps patients choose a doctor for online consultation to receive expert advice based on their condition. Simultaneously, most of the active doctors on the website have access to the Popular Science zone in which doctors can share relevant health information with patients by publishing popular science articles. The platform has a large pool of quality doctors. Based on the large number of users and active doctors, we chose this platform as the data source for this study. Following interviews with specialists in different departments, we deduced that cancer, cardiovascular diseases, and psychological diseases are the types of ailments that are currently witnessing an increase in consultations. Therefore, based on expert recommendations, this study collected data for analysis on four diseases: lung cancer, brain hemorrhage, hypertension, and depression. Starting in March 2022, we collected data every two weeks for a total of four data collections involving 4500 physicians. After excluding missing values and data of physicians who did not publish scientific articles and physicians who published less than or equal to five articles, we finally obtained the personal characteristics data of 1295 physicians and their published popular science articles. The popular health science articles studied in this paper were short popular science articles on topics such as disease treatment, disease science, disease prevention, and health knowledge of different diseases published by doctors themselves.

### 3.3. Variable Description and Measurement

The descriptions of the variables involved in this study and the specific measures are shown in Table 1.

#### 3.3.1. Physician Performance

Physicians’ performance in this study is defined as the financial return that physicians receive from the platform, measured by a combination of the number of visits and the consultation cost. According to our prior use and observation of the online medical platform, we determined that the cost of doctor consultation is mainly divided into three types: graphic consultation, one question and one answer, and telephonic consultation. Of these, graphic consultation and telephonic consultation are the main consultation methods provided by most doctors. As the time unit charged by each doctor for the graphic consultation is not uniform, this study chose the cost for telephonic consultation as a measure of the price factor of the doctor’s performance. As a typical telephonic consultation lasts 10–20 minutes, we use the calculated minute rate as the average consultation price per physician. 

Due to the large differences in the financial rewards that different physicians receive from online medical platforms, the results of subsequent regression analyses may be abducted by high-performing physicians, while the relevant characteristics of lower-performing physicians are not fully represented in the regression analysis. Furthermore, the order of magnitude difference between the dependent variable physician performance and the independent variable science article characteristics in this study is also large. We find that the logarithmic function in mathematical functions can be considered as a monotonic transformation that does not change the relative size of the original data, and taking logarithms itself does not change the correlation between variables [54]. Therefore, for the above reasons, in this study, to avoid parameter estimates being abducted by individual extreme outliers during regression, the product of price rates per physician and total number of patients were taken as logarithms when measuring physician performance. The formula for calculating the physicians’ performance is shown below:

(1) $$performance_{i}=\ln\left(price_{i}*patients_{i}\right)$$

$price_{i}$ is expressed as the doctor’s rate per minute of telephone consultation. $patients_{i}$ is expressed as the total number of patients the physician has in the online healthcare platform.

#### 3.3.2. Topic Focus

This study measured topic focus by mining the topics contained in physician-published popular science articles and the standard deviation of the probability of topic distribution. Latent Dirichlet allocation (LDA) topic modeling is a traditional text analysis technique that mines text topics by extracting keywords and is widely used in various fields [55]. The LDA model is a mathematical model proposed by Blei et al. in 2003 to infer the topic distribution of a document [56]. It is essentially a three-layer Bayesian probabilistic model, with the three layers referring to the three-layer structure of words, topics, and documents [57]. The purpose of LDA is to identify the topics in a document, i.e., to turn the document-vocabulary matrix into a document-topic matrix and a topic-vocabulary matrix. The probability of generating topics per document and the probability of generating words per topic can be derived by solving the matrices. Therefore, in this study, we used LDA to analyze the topics of popular science articles on different diseases written by each physician. As the title of an article is a reflection of the core content of an article, we combined all the titles of popular science articles into one long text and performed LDA topic analysis with individual physicians.

The key to applying LDA subject modeling is to first select the appropriate number of subjects. In this paper, we chose perplexity as the evaluation index, which is a common index used to evaluate the predictive ability of LDA models. Therefore, the perplexity is chosen to determine the optimal number of subjects in this study. A smaller value of perplexity means that the classification of the model is better, and the formula for calculating the perplexity is:

(2) $$perplexity\left(D\right)=\exp\left|-\frac{\sum_{d=1}^{M}\log p\left(w_{d}\right)}{\sum_{d=1}^{M}N_{d}}\right|$$

where *D* denotes the test set in the corpus, *M* denotes the number of documents in the test set, $N_{d}$ denotes the number of words in the *d*-th document, $w_{d}$ denotes the words in document d, and $p\left(w_{d}\right)$ denotes the probability of word $w_{d}\;$in the *d*-th document.

**Figure 2:**
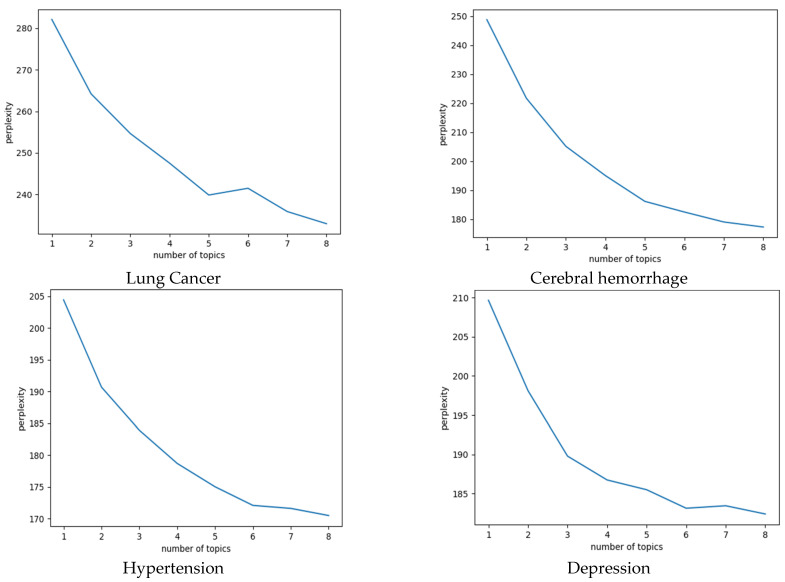
Perplexity Curve.

In this study, we use scikit-learn in the python toolkit to extract topics from physician science articles under each of the four diseases, and select the optimal number of topics based on the perplexity curve shown in Figure 2. We set the optimal number of topics for lung cancer, brain hemorrhage, hypertension, and depression to 5, 5, 6, and 6, respectively. We found that when the number of topics was set to 5 and 7 for lung cancer and brain hemorrhage, there was little difference between the perplexity values. However, the curve trended more flatly after 7. Then, we compared the visualizations again and inferred that there were overlapping topics when the number of topics was set to 7. Therefore, we finally set the optimal number of topics for both to 5.

The final probability distribution of each topic was obtained for each doctor under the corresponding text. It has been demonstrated that the standard deviation is the most common indicator of the dispersion of a dataset, with a large standard deviation indicating that most of the values differ from the mean and a small standard deviation indicating that the values are closer to the mean [58]. To measure the topic focus of the popular science articles in this study, we assume that when the standard deviation of the probability of topic distribution is large, most of the popular science articles written by physicians are concentrated on certain topics and the topic focus is strong, whereas when the standard deviation of the probability of topic distribution is small, the popular science articles written by physicians are scattered over various topics and the topic focus is weak. Finally, the standard deviation of the probability of topic distribution for popular science articles written by each physician was used as an indicator of the topic focus.

#### 3.3.3. Readability

As article titles help readers obtain key information from articles, this paper measures readability through popular science article titles. The most commonly used tool to assess the readability of materials is the readability formula, which focuses on the complex vocabulary, sentences, and number of words contained in the material to determine whether the information is comprehensible. The English readability formula has been widely used in the medical field; however, as the Chinese readability formula has not been widely used, this study considers the English readability formula and replaces the number of words in the formula with the number of words in Chinese with reference to existing studies [59]. Authors previously have highlighted that words with a high number of letters are not necessarily complex words; if these words are commonly used in society, they will no longer have complexity [60]. In summary, this paper defines complex words as words whose meaning is difficult to understand. The simple measure of gobbledygook (SMOG) readability formula is considered as the best in the medical field when evaluating differences in the application of different readability formulas to health materials. Therefore, this study used the SMOG formula to measure the readability of popular science article titles by physicians. The formula is specified as follows:

(3) $$readability=1/\left(1.043*\sqrt{number\;of\;complex\;words}+3.1291\right)$$

The key to the readability formula used in this study is the calculation of the number of complex words in the title of the article. Davison and Kantor suggested that the background knowledge of the target audience group is more important than attempting to make the text conform to the formula definition of readability [61]. In the context of this paper, the audience group for popular science articles by physicians is general users who are not medical professionals. Based on pre-interviews of 10 general users who perceived difficult words in reading science articles, we determined that the difficult words for general users were generally rare medical specialties, such as complex drugs and disease names. Previous studies have used medical dictionaries to learn medical vocabulary in texts [62]. Conversely, the focus in this study’s scenario was on the complexity of the medical vocabulary, and whether it was a complex medical vocabulary was a subjective judgment of the patient, rather than a medical vocabulary as defined in the medical professional field. For example, patients do not consider words such as hypertension, insulin, and autism to be difficult medical words as these medical terminologies have been widely disseminated and popularized. In summary, this study used a manual tagging method to screen complex medical vocabulary in the titles of popular science articles and used the jieba library to split the Chinese words in the titles of the articles and removed the dummy and meaningless words.

Venugopal, G., Pramod, D., and Saini, J.R. proposed three ways to mark complex words and stated that using a Likert scale to mark complex words would be more unbiased [63]. On the scale, 1 indicates very complex words, whereas 5 indicates very simple words; when the score is less than or equal to 3, words are marked as complex. This method was adopted in this study to mark complex words manually in physicians’ popular science articles. First, four nonmedical volunteers were invited to mark the words that were difficult to understand in the result of the division of the title of the popular science article, and then the screened words were designed as a word complexity scale, which was distributed to 30 nonmedical volunteers. The Likert scale was used to obtain a complexity score for each word, and the words with an average score of less than or equal to 3 were finally marked as complex words. Based on the aforementioned work, we calculated the number of complex medical words contained in the title of each popular science article to obtain the readability value of each article and then used the average value of the readability of all the popular science articles of each physician as the readability value of the physician’s popular science articles.

#### 3.3.4. Form Diversity

In this paper, we divided the presentation forms of popular science articles into three categories, text, voice, and video, and calculated the number of physicians’ popular science articles containing forms with a minimum of 1 and a maximum of 3. Format diversity was measured by counting the non-repeated counts of physicians’ popular science article forms. For example, if Dr. Wang publishes a total of 10 popular science articles in different formats, including audio (1), videos (4), and texts (5), then Dr. Wang’s form diversity is 3. Dr. Li publishes a total of 50 articles, including 20 texts and 30 videos; thus, Dr. Li’s form diversity is 2.

#### 3.3.5. Other Variables

For the control variables, article count of likes (LikeCnt) is measured by the average of likes count of all popular science articles for each physician. The number of years of online service is categorized by year, 1 being the year 2021 for the platform, 14 being the year 2008 for the platform; the larger the number, the longer the doctor has been serving on the online medical platform. The city level refers to the classification provided by the First Financial Weekly, 3 being a first-tier city, 2 being a new first-tier city, and 1 being a second-tier city; the larger the value, the higher the city level. The degree of disease urgency is classified according to the degree of urgency for immediate treatment, with cerebral hemorrhage representing high disease urgency, recorded as 2, and hypertension, lung cancer, and depression representing low disease urgency, recorded as 1. Hospital rank is classified according to whether it is a tertiary care hospital, where 1 is not a tertiary care hospital and 2 is a tertiary care hospital. Physician titles are classified according to chief physician, deputy chief physician, and other physicians, with 3 being the highest title and 1 being the lowest title.

## 4. Results

### 4.1. Descriptive Statistical Analysis

Table 2 provides the descriptive statistics of the number of physicians and popular science articles on each type of disease. A comprehensive analysis revealed that the highest number of popular science articles was related to cerebral hemorrhage, accounting for 35.6% of the total number of articles. By comparing the number of articles per capita, we deduced that doctors specialized in treating cerebral hemorrhage were more active in sharing popular science knowledge, whereas doctors specialized in treating hypertension and depression were relatively less active in publishing popular science articles.

Table 3 offers the results of descriptive statistics for all the variables involved in this study. The average value of doctors’ city rank is 2.302, indicating that most of the platform’s doctors come from first-tier cities in China, and online medical care is more popular in high-ranking cities. The average number of years of online service for doctors is 9.743, indicating that most doctors have been on the platform earlier and have been using it for many years. The difference between the maximum and minimum values of the likes of the article is significant, which indicates a large difference in the popularity and dissemination of popular science articles.

### 4.2. Correlation Analysis

We performed correlation analysis on the independent and control variables to test for the presence of multicollinearity between the variables. As can be observed in Table 4, the correlation coefficients between the variables are less than 0.5; thus, there is no serious multicollinearity.

Variance inflation factor (VIF) is a measure of the severity of multicollinearity in a multiple linear regression model. It represents the ratio of the variance of the estimated regression coefficients to the variance of the regression coefficients between the independent variables that are not linearly correlated [64]. Therefore, in this paper, the VIF test was conducted for each variable. As shown in Table 5, the VIF values of all variables were less than 5, further confirming the absence of multicollinearity.

### 4.3. Regression Results

This study used mixed OLS regression to estimate the panel data and the regression results are shown in Table 6.

Model 1 included only control variables, and the results revealed that city class, years of online service, number of likes, and physicians’ title had a significant positive impact on physicians’ performance, whereas disease urgency had a significant negative impact on physicians’ performance. Typically, a higher city rank has more medical resources and a higher average level of physicians; thus, city rank had a positive effect on physicians’ performance. The longer the number of years of online service, the more experienced the doctor is for online consultation service; this result is consistent with the purpose of offline consultation, and patients are inclined to choose doctors who work longer. The more likes the article has, the more popular the doctor’s popular science articles are and the more patients trust the doctor’s expertise, thus, contributing positively to the doctor’s performance. The higher the doctor’s title level, the stronger the doctor’s expertise, and patients are more inclined to choose a doctor with a higher title level for consultation. For diseases of high urgency, which require immediate treatment and cannot be delayed, patients need to be treated promptly at offline hospitals and may not be inclined to consult through online medical platforms. Patients with less urgent diseases not requiring immediate surgical intervention and not having a strict timeframe for treatment may prefer to be pre-screened through online medical platforms to acquire a general idea of their disease status. Therefore, the lower the level of disease urgency, the better is the physicians’ performance.

Model 2 adds the explanatory variable of topic focus to model 1, and the result shows a significant positive impact of topic focus on physicians’ performance, β = 7.625, *p* < 0.001, and H1 is validated. The more physicians publish popular science articles focused on one or a few topics, the better the physicians’ performance.

Model 3 adds readability as an explanatory variable to model 2, and the result shows a significant positive impact of readability on physicians’ performance, β = 1.530, *p* < 0.001, and H2 is validated. The less complex medical vocabulary included in the topics of the popular science articles published by doctors, the easier it is for patients to understand and the more beneficial the popular science articles are for patients. When patients perceive that they have gained popular health science knowledge, they may increase their trust in doctors, which, in turn, helps promote the improvement of doctors’ performance.

Model 4 adds the explanatory variable of form diversity to model 3, and the result shows a significant positive impact of form diversity on physicians’ performance, β = 0.271, *p* < 0.001, and H3 is validated. The more diversified the presentation format of doctors’ popular science articles, the more patients may be inclined to think that doctors exert more effort into their science numbers and operate more attentively compared to other doctors. Applying different media formats may meet different reading needs of patients, which will leave a better impression of the doctor and, thus, promote the doctor’s performance.

## 5. Robustness Test

To enhance the reliability of the study’s findings, the paper chose other methods to measure the three independent variables of topic focus, readability, and form diversity to verify whether consistent conclusions could be drawn.

(1) Alternative Measurement Method of Topic Focus

When we analyzed the topics by disease type, we deduced that there was a crossover in the topics included in the popular science articles under different diseases. For example, the popular science articles on hypertension and depression included the same topics of diabetes and children. Therefore, all popular science articles included in the four diseases are presently combined for LDA topic analysis, and the optimal number of themes is 6 according to the perplexity curve. Then, the standard deviation is calculated for the topic distribution probability, which is another measure of topic focus.

(2) Alternative Measurement Method of Readability

The SMOG index was used as a formula to measure the readability of popular science articles, and studies have shown that SMOG and gunning fog index (GFI) are the best choices to measure the readability of online health information [65]. The GFI is at present used to measure the readability of popular science articles for robustness testing, and the formula is specified as follows:

(4) $$readability=1/\left(0.4*\frac{number\;of\;words}{number\;of\;sentences}+100*\frac{number\;of\;complex\;words}{number\;of\;words}\right)$$

As the readability of this article is measured by the titles of popular science articles, the number of sentences defaults to 1.

(3) Alternative Measurement Method of Form Diversity

The variable of diversity is most commonly used in biology to evaluate the amount of species diversity in a community, and studies have summarized common formulas for measuring species diversity [66]. We believe that in this study a doctor can be equivalent to a community, and the three media—text, voice, and video—can be considered three species in a community. In summary, we believe that it is feasible to apply the formula for measuring species diversity in this study. The Shannon diversity index is used here to assess the diversity of popular science article formats. The specific formula is as follows:

(5) $$Form\;Diversity=-\sum \left(p_{i}*\ln p_{i}\right)$$

$p_{i}\;$indicates the ratio of the number of articles of the *i*th form to the total number of articles for each physician.

The regressions were conducted again based on the above three methods of replacing the measures of topic focus, readability, and form diversity. The regression results are shown in Table 7, which are consistent with the regression results in Table 6, indicating that the regression results of this study are robust and the conclusions are reliable.

## 6. Discussion

### 6.1. Key Findings

This study investigates the effect of the characteristics of popular science articles on physicians’ performance. The study results revealed that the topic focus of articles positively affects physicians’ performance, and the clearer the topic of an article posted by a physician, the stronger the positive effect on their performance. Patients can judge a doctor’s professionalism through their popular science articles. When most of the doctor’s popular science articles are about the same topic of health knowledge, it indicates that the higher the doctor’s knowledge in this field, the higher the patient’s trust in them will be. Thus, the topic focus of popular science articles has a positive impact on physicians’ performance.

The readability of popular science articles has a positive impact on physicians’ performance. The less complex the medical vocabulary a doctor’s article contains, the more readable the article is and the easier it is for patients to obtain the right health information from it. When patients benefit from the doctor’s popular science articles, they think that the doctor is more likely to solve their disease confusion and may be more inclined to choose that doctor for consultation. Thus, physicians with highly readable popular science articles are more likely to achieve high performance.

The variety of popular science article formats positively contributes to physicians’ performance. Different media presentation methods have their different advantages and disadvantages, and when popular science articles are presented in more diverse forms, they may be able to meet the needs of different patients. When patients see the popular science articles presented through different forms before reading, they will first think that the doctor uses the popular science zone more often and may be relatively more active on the platform based on the surface characteristics, and thus, will have a better initial impression of the doctor. In this manner, physicians with a greater diversity of article formats may have higher performance.

### 6.2. Research Contribution

#### 6.2.1. Theoretical Implications

This study provides theoretical contributions mainly in the following aspects:

(1) This paper enriches the study of factors influencing physicians’ performance. It focuses on the feature of a popular science section in an online healthcare platform and examines what specific characteristics of popular science articles would benefit physicians’ performance. Previous studies of popular science articles have extracted only superficial characteristics, such as the number of articles, article readership [67], research on popular science articles has not been conducted in sufficient depth, and features related to the content of the articles are rarely mentioned. This paper further explores the content characteristics per se of science articles through thematic and readability analyses and verifies their positive impact on physicians’ performance, thereby expanding the research on the impact on physicians’ performance.
(2) This study proposes a new influence mechanism for health science articles. A majority of the previous studies have focused on the motivation of sharing health science popularization knowledge, whereas a few studies have analyzed the utility of health science popularization knowledge features by considering science popularization behavior as an independent variable. In this paper, three characteristics of popular science articles (thematic relevance, readability, and formal variety) are used as independent variables to explore their influence mechanisms, thus proposing a new link between characteristics of popular science articles and physicians’ performance.

#### 6.2.2. Practical Implications

The findings of this paper have practical implications for all three parties (physicians, platforms, and patients) of online healthcare platforms.

(1) This study has important implications for how doctors can publish popular science articles more effectively in the near future. In terms of topics, doctors should place greater emphasis on article content when publishing popular science articles and focus on health information pertaining to their own areas of expertise rather than a broad range of popular science knowledge. Simultaneously, care should be taken to consider patients’ ability to accept medical expertise, and excessive medical jargon, such as drug names and treatments, which may confuse patients, should be avoided. Finally, doctors can choose from the appropriate media forms to publish articles according to the varied contents of science popularization. For example, they can choose video publication when they need to demonstrate actions and choose graphic publication when they need to summarize precautions.
(2) This paper also has implications for the future sustainability of online healthcare platforms. This study finds that the relevance, readability, and variety of formats have a positive impact on doctors’ performance, and that patients’ demand for popular health knowledge is growing, and online health knowledge popularization has become a major trend. The platform can encourage doctors to participate more in publishing popular science articles to improve their performance, stabilize the main body of doctors on the platform, and make the module of doctors’ popular science one of the key focuses of future development, which will help the platform prosper and develop continuously.
(3) This study also has realistic significance for patients. According to the findings of this study, the patients focused on the topic and readability of the content and the form diversity of the articles when they read the doctors’ popular science articles. The more targeted the content of the doctor’s popular science articles is, the more patients can clearly grasp the doctor’s area of expertise; the more readable the popular science articles are, the more the patients can correctly understand the popular science knowledge regarding health. Therefore, this study has practical implications for patients in terms of both choosing doctors and effectively grasping popular science knowledge regarding health.

### 6.3. Limitations

There are some limitations in this study. First, only four diseases were considered in the dataset of this study: lung cancer, brain hemorrhage, hypertension, and depression. Although representative diseases were selected based on expert opinion, all diseases were not covered, and future studies should consider other kinds of diseases to verify whether the findings are consistent. Second, the variable of topic focus proposed herein is a combination of multiple diseases. The heterogeneity of the impact of specific topic content on physicians’ performance can be explored in the future depending on the type of disease.

## 7. Conclusions

Based on the ELM model, this study explored the impact of specific characteristics of physicians’ popular science articles published in online medical platforms on physicians’ performance in terms of content characteristics (topical focus and readability) and form characteristics (form diversity). The results showed that topic focus had a positive impact on physician performance, and patients perceived that physicians’ posting of disease- or domain-specific health science knowledge was more likely to highlight their own expertise. Readability positively influenced physician performance, and physicians should take into account patients’ health literacy when publishing popular science articles and avoid excessive medical jargon in the articles to affect patients’ reception. Form diversity has a positive effect on physicians’ performance. Physicians can choose different media presentation formats for different features of science popularization knowledge when publishing science popularization articles, so that patients can perceive physicians’ commitment in the science popularization area and increase their trust in them, which is conducive to physicians’ performance. The findings of our study are important for guiding doctors to publish popular science articles in the future so as to improve their performance more effectively. The platform can also use the findings of this study as a reference to further optimize the platform’s science popularization area module to help patients obtain health science knowledge more effectively.

## Figures and Tables

**Table 1:** Variable Description and Measurement.

	Name	Description	Measurement
**Dependent** **variable**	Performance	The financial rewards doctors receive from the platform	Telephone consultation rate per minute * total number of patients then logarithm
**Independent variable**	Topic Focus	The extent to which physicians publish popular science articles focusing on a particular field or topic	The probability of distribution of all popular science articles of the doctor under each topic, calculate the standard deviation of the probability
	Readability	Physicians publish popular science articles that are easy for patients to understand and grasp	Screening articles for complex medical vocabulary and calculating them by readability formulas
	Form Diversity	Physicians publish popular science articles in a variety of presentation formats, including text, audio, and video	text; 2—voice; 3—video All popular science articles by doctors are counted non-repeatedly according to the above three

**Table 2:** Descriptive Statistics for Physicians and Popular Science Articles.

Disease	Number of Physicians	Number of Articles	Average Article Count	Percentage of Articles
Lung Cancer	344	27,151	78.927	28.8%
Cerebral hemorrhage	404	33,605	83.181	35.6%
Hypertension	258	16,168	62.667	17.1%
Depression	289	17,505	60.571	18.5%
Total	1295	94,429	285.346	100.0%

**Table 3:** Results of Descriptive Statistics of Variables.

Variable	N	Mean	Median	Std.Dev.	Min	Max
Performance	5180	9.714	9.858	1.825	3.219	14.94
City	5180	2.302	2	0.765	1	3
Duration	5180	9.743	10	3.318	1	14
LikeCnt	5180	5.501	1.5	13.22	0	161.2
Urgency Level Doctorgrade	5180 5180 5180	1.312 1.961 2.574	1 2 3	0.463 0.195 0.607	1 1 0	2 2 3
Topic Focus	5180	0.082	0.064	0.061	0.003	0.387
Readability	5180	0.303	0.315	0.0380	0.0840	0.540
Form Diversity	5180	1.352	1	0.537	1	3

**Table 4:** Correlation Analysis.

	City	Duration	LikeCnt	Urgency	Level	Doctorgrade	Topic	Readability	Form
City	1								
Duration	0.113	1							
LikeCnt	0.0743	−0.0667	1						
Urgency	−0.0675	0.0291	−0.0914	1					
Level	−0.0239	−0.0205	0.0416	−0.0522	1				
Doctorgrade	−0.0971	0.406	−0.125	−0.0461	−0.0114	1			
Topic	0.0673	0.171	−0.0402	0.149	−0.0518	0.0812	1		
Readability	−0.0028	−0.0258	0.0489	−0.444	0.0201	0.0154	−0.136	1	
Form	0.0612	−0.0789	0.197	−0.0195	0.0161	−0.111	0.285	0.00530	1

**Table 5:** VIF Test.

Variable	VIF	1/VIF
Urgency	1.28	0.778
Duration	1.27	0.787
Doctorgrade	1.26	0.796
Readability	1.25	0.797
Topic Focus	1.19	0.842
Form Diversity	1.17	0.853
LikeCnt	1.07	0.932
City	1.06	0.946
Level	1.01	0.993
Mean VIF	1.17	

**Table 6:** Regression Results.

Variable	Dependent Variables: Performance			
	Model 1	Model 2	Model 3	Model 4
City	0.430 ***	0.391 ***	0.393 ***	0.388 ***
	(0.057)	(0.055)	(0.055)	(0.055)
Duration	0.125 ***	0.105 ***	0.105 ***	0.109 ***
	(0.015)	(0.015)	(0.015)	(0.015)
LikeCnt	0.035 ***	0.036 ***	0.036 ***	0.033 ***
	(0.004)	(0.004)	(0.004)	(0.004)
Urgency	−1.027 ***	−1.175 ***	−1.120 ***	−1.108 ***
	(0.105)	(0.101)	(0.104)	(0.103)
Level	0.164	0.257	0.259	0.243
	(0.228)	(0.216)	(0.216)	(0.214)
Doctorgrade	0.152 *	0.127	0.127	0.146 *
	(0.084)	(0.081)	(0.081)	(0.082)
Topic		7.622 ***	7.688 ***	6.896 ***
		(0.708)	(0.707)	(0.736)
Readability			1.546 ***	1.461 ***
			(0.521)	(0.523)
Form				0.281 ***
				(0.075)
_cons	7.945 ***	7.683 ***	7.129 ***	6.789 ***
	(0.532)	(0.502)	(0.532)	(0.538)
N	5180.000	5180.000	5180.000	5180.000
r2	0.249	0.311	0.311	0.317

Standard errors in parentheses * *p* < 0.1, ** *p* < 0.05, *** *p* < 0.01.

**Table 7:** Regression Results.

Variable	Dependent Variables: Performance			
	Model 1	Model 2	Model 3	Model 4
City	0.430 ***	0.407 ***	0.429 ***	0.423 ***
	(0.057)	(0.055)	(0.055)	(0.055)
Duration	0.125 ***	0.101 ***	0.101 ***	0.104 ***
	(0.015)	(0.015)	(0.015)	(0.015)
LikeCnt	0.035 ***	0.036 ***	0.036 ***	0.035 ***
	(0.004)	(0.004)	(0.004)	(0.004)
Urgency	−1.027 ***	−0.926 ***	−0.879 ***	−0.887 ***
	(0.105)	(0.101)	(0.103)	(0.103)
Level	0.164	0.312	0.318	0.323
	(0.228)	(0.218)	(0.220)	(0.218)
Doctorgrade	0.152 *	0.181 **	0.183 **	0.190 **
	(0.084)	(0.082)	(0.082)	(0.082)
Topic1		6.861 ***	7.300 ***	7.002 ***
		(0.611)	(0.615)	(0.627)
Readability1			0.620 **	0.588 **
			(0.240)	(0.242)
Form1				0.401 **
				(0.185)
_cons	7.945 ***	7.021 ***	6.488 ***	6.460 ***
	(0.532)	(0.516)	(0.554)	(0.550)
N	5180.000	5180.000	5180.000	5180.000
r2	0.249	0.309	0.313	0.315

Standard errors in parentheses * *p* < 0.1, ** *p* < 0.05, *** *p* < 0.01

## Data Availability

Data sharing is not applicable to this article.
